# The Role of Chemotherapy in Upper Tract Urothelial Carcinoma

**DOI:** 10.1155/2009/419028

**Published:** 2009-01-26

**Authors:** Peter H. O'Donnell, Walter M. Stadler

**Affiliations:** Section of Hematology/Oncology, Department of Medicine, University of Chicago, Maryland Avenue, MC2115, Chicago, IL 60637, USA

## Abstract

Locally advanced upper tract urothelial carcinoma has a poor prognosis. While surgery represents the only potentially curable therapeutic intervention, recurrences are common and typically systemic in nature. It is thus reasonable to consider perioperative chemotherapy in an effort to decrease the risk of recurrence. There are very little direct data providing clinical guidance in this scenario. For urothelial cancer of the bladder, there are randomized phase III data demonstrating a survival advantage with neoadjuvant cisplatin-based combination chemotherapy. Although arguments favoring adjuvant chemotherapy could be made for upper tract urothelial cancer, the loss of renal function that occurs with nephrectomy can complicate administration of appropriate perioperative treatment. Therefore, by analogy to urothelial carcinoma of the lower tract, it is argued that cisplatin-based neoadjuvant chemotherapy should be the standard of care for patients with locally advanced upper tract urothelial cancer.

## 1. Introduction

Locally advanced upper tract urothelial carcinoma has a poor prognosis. Surgical series suggest that, notwithstanding nodal status, the disease-specific five-year survival rates for stages T2 and T3 disease are 73% and 40%, respectively, while the median survival for T4 patients is approximately 6 months [1]. Importantly, the vast majority of patients with invasive upper tract urothelial carcinoma have stage T3 or greater disease at the time of surgery [2, 3], and, if investigated, at least 20–25% will have lymph node involvement at the time of surgery [4–6]. The poor prognosis is furthermore reflected by mortality estimates in which the mortality to incidence ratio for upper tract disease is approximately 0.34 [7], whereas for lower tract urothelial cancer it is 0.20 [8]. This may be due in part to the notorious difficulty of diagnosing earlier-stage urothelial cancer of the upper tract.

The most common presenting symptom of upper tract urothelial carcinoma, like its bladder counterpart, is hematuria [1]. Unfortunately, urine cytology is not particularly sensitive for diagnosing urothelial carcinoma of any location [9, 10]. Anterior grade and retrograde pyelogram, or ureteroscopy with visualization of the renal pelvices, are technically challenging and not routinely performed in the evaluation of hematuria. Computed tomography (CT) imaging is also not very sensitive for early stage disease [11, 12].

While surgery represents the only potentially curable therapeutic intervention for upper tract urothelial cancer, systemic recurrences are common [1, 3, 13]. It is thus reasonable to consider perioperative chemotherapy in an effort to decrease the risk of recurrence. Unfortunately, with only approximately 2000 cases annually in the United States [7, 14], and with the historical difficulties in accruing urothelial carcinoma patients to clinical trials, there are very little direct data providing clinical guidance in this scenario. Using analogy to urothelial carcinoma of the lower tract, we believe that cisplatin-based neoadjuvant chemotherapy should be the standard of care for patients with locally advanced upper tract urothelial cancer.

## 2. Is Urothelial Cancer of the Upper Tract Different?

Bladder and upper tract urothelial carcinomas have traditionally been considered separate diseases in the urologic and surgical literature mainly because the surgical approach to these diseases is so different. Yet, recent evidence has suggested that the anatomic location of disease has no bearing on tumor behavior, in that recurrence and mortality rates from upper and lower tract carcinomas are similar when adjusted for tumor stage and grade [15]. Additionally, from a biologic prospective, there is very little difference between urothelial carcinomas that arise from these different sites. First of all, the most important epidemiologic risk factor for urothelial cancer remains exposure to tobacco products [16, 17], and this is true regardless of the site of origin. Secondly, the urothelial tissue itself is histologically indistinguishable by site [18]. Certainly, there are differences in the underlying stroma and supportive tissue, but the importance of these differences in the treatment of systemic disease, including the microscopic systemic disease that is being targeted in perioperative therapy, is debatable. Thirdly, the molecular oncogenic events appear to be the same between upper and lower tract urothelial cancers. For example, for both origins, chromosome 9 deletions are the most common genetic abnormality [19, 20], and chromosome 9 and p53 alterations appear to be present at similar frequencies in upper and lower tract lesions [20, 21]. Fourth, it has been the practice of the medical oncology community to include urothelial carcinoma patients in trials of metastatic disease, regardless of the site of origin. A number of large studies have thus included at least a fraction of patients whose initial tumor began in the upper tract [22–28]. In none of those studies was the site of origin an important prognostic factor in the context of systemic chemotherapy. Finally, the high incidence of secondary (40–50%), or, less commonly, synchronous (8%) lower tract disease in patients with upper tract disease [1, 29] also supports the notion that these cancers actually represent one disease process in such patients. It is thus reasonable to consider systemic and perioperative chemotherapy for locally advanced upper tract urothelial cancer by analogy to urothelial cancer of the bladder.

For urothelial cancer of the bladder, there are randomized phase III data demonstrating a survival advantage with neoadjuvant cisplatin-based combination chemotherapy. The two largest trials were conducted by an international collaboration [30] and by Grossman et al. [31]. A meta-analysis including all neoadjuvant chemotherapy trials confirms the conclusion [32]. Others have argued that the absolute benefit of neoadjuvant chemotherapy is modest [33], and that the more accurate clinical staging afforded by surgery [34] allows better patient selection on the basis of prognostic factors [35]. Under the assumption of equivalent benefit irrespective of underlying prognosis, the absolute benefit of perioperative chemotherapy for poor prognosis patients is certainly going to be greater than that for good prognosis patients. Nevertheless, the randomized studies of adjuvant chemotherapy in urothelial cancer of the bladder have been undersized and underpowered for detecting a clinically significant benefit [36–40].

Although similar arguments favoring adjuvant chemotherapy could be made for upper tract urothelial cancer, the loss of renal function that occurs with nephrectomy can further complicate administration of appropriate perioperative treatment. In this regard, the above noted meta-analysis [32] demonstrated survival benefits with neoadjuvant therapy only when cisplatin-based combination chemotherapy was utilized, and two randomized studies in the metastatic setting have strongly suggested that carboplatin, which is typically substituted for cisplatin in patients with renal dysfunction, is an inferior agent [41, 42]. Furthermore, the decrement in renal function associated with nephrectomy is not inconsequential, as evidenced by studies in renal cancer patients undergoing nephrectomy [43, 44]. This may be even more important in urothelial cancer patients who are often smokers and have other smoking-related comorbidities. Finally, anecdotal experience and early evidence [45, 46] suggest that even among patients in whom neoadjuvant chemotherapy is indicated, only a minority actually receive chemotherapy. This raises concerns about a lack of adherence to chemotherapy recommendations [47] in the urologic community, or perhaps an unwillingness by patients to be treated. Even if some urologists forgo neoadjuvant chemotherapy referral in favor of future adjuvant administration, adherence percentages in the postoperative setting are likely to be even lower, because of both patient and surgeon factors [48, 49]. Furthermore, despite hypothetical concerns about the potential for increased surgical morbidity after neoadjuvant chemotherapy, the data in bladder cancer patients have strongly demonstrated that this does not occur [49, 50], so we anticipate that the same would be true for neoadjuvant chemotherapy in upper tract disease.

## 3. Conclusions

Upper tract urothelial carcinoma, when not metastatic, typically presents with locally advanced disease. Such disease has a poor prognosis because of the high risk of systemic recurrence. Although the surgical approach to upper tract and lower tract urothelial cancers is markedly different, the biology of these diseases is for the most part indistinguishable. Certainly, the response to therapy appears to be the same. Given the rarity of upper tract urothelial cancer and the difficulty of accruing to clinical trials, recommendations for perioperative chemotherapy must currently be based on similarities to its lower tract counterpart. In this regard, neoadjuvant chemotherapy is the standard of care based on improvements in survival in well-conducted phase III trials. Such data does not exist with adjuvant therapy in bladder cancer, and adjuvant therapy for upper tract disease is further complicated by the difficulty of administering cisplatin-based regimens to patients who may suffer a decrement in renal function following nephrectomy. Therefore, until and unless specific trials are conducted, the most reasonable standard for locally advanced upper tract urothelial cancer is neoadjuvant, cisplatin-based combination chemotherapy prior to nephrectomy and surgical resection.
